# Supplementary material for: Metasurface-Based Quantum Searcher on a Silicon-On-Insulator Chip
Source: Micromachines (Basel). 2022 Jul 28;13(8):1204. doi: 10.3390/mi13081204 (PMC9413265; doi:10.3390/mi13081204)
Supplement: Supplementary file 1 [file micromachines-13-01204-s001.zip › micromachines-1829112-supplementary.pdf]

Supplementary Materials

S1. Designing of the quantum searcher in free space

In order to compare with the on-chip quantum searcher, we performed a numerical simulations of the free-space equivalent medium quantum searcher using Gallop, a three-dimensional electromagnetic field simulation software. Gallop is a large-size electromagnetic field simulation and optimization software based on an efficient electromagnetic modeling theory combined with multi-algorithmic hybrid techniques. In this work, the electromagnetic field simulation function in Gallop is used to calculate photonics problems with the help of finite difference time domain (FDTD) method. The simulation domain was 3D, rectangular and non-uniformly gridded. The minimum mesh step was set to 50 nm and the simulation time was set to 2000 fs to obtain accurate simulation results. Periodic boundary conditions were applied in the z-direction, while perfectly matched layer-absorbing (PML) boundary conditions were applied in the x- and y-directions.

We implement the quantum search algorithm in the classical realm based on the equivalent medium. The electric field amplitude of the incident microwave "E(y)" is recognized as the probability amplitude of the equivalent quantum state. Spatial positions "y" are used to label the items in the database. The maximum number of the database is fixed by the full width at half-maximum (FWHM) "D" of the incident intensity profile with near-Gaussian distribution. In Table S1, we compare the general protocols for performing quantum search algorithm in the quantum and classical realm to clarify the differences between the two strategies.

Table S1. The general protocol of performing quantum search algorithm with metamaterial in quantum and classical realm.

Quantum		Classical
Items in the database $\|\boldsymbol{i}\rangle$		"y"
Probability amplitude of the equivalent quantum state		"$E(y)$"
The maximum number of the database $\boldsymbol{N}$		"D"
$\boldsymbol{U_m}$	$I - 2\|s\rangle\langle s\|$	$exp\left(i2\pi\sqrt{\varepsilon_m(y)}\, d_m/\lambda_0\right)$
$\boldsymbol{IAA}$	$H^{\otimes n}$<br>$I - 2\|0\rangle\langle 0\|$<br>$H^{\otimes n}$	F<br>$exp\left(i2\pi\sqrt{\varepsilon_0(y)}\, d_0/\lambda_0\right)$<br>$F$

In Figure S1a, we give a schematic diagram of the quantum searcher based on the equivalent medium. In order to facilitate the simulation, we set the magnetic permeability $\mu = \mu_0$ for the whole system. In this case, the effective permittivity of the $U_m(U_0)$ and GRIN subblocks, $\varepsilon_{m(0)}$ and ε_g, can be expressed as:

$$\varepsilon_{m(0)}(y) = \begin{cases} \left(\dfrac{1.25\lambda_0}{d_{m(0)}}\right)^2, & 0.5\,\pi \\ \left(\dfrac{\lambda_0}{d_{m(0)}}\right)^2, & 0 \end{cases}, \quad for\ U_{m(0)} \tag{S1}$$

$$\varepsilon_g(y) = \varepsilon_c[1 - (\pi/2l)^2 y^2], \quad for\ GRIN \tag{S2}$$

where λ_0 is the wavelength of the incident beam in vacuum, $d_{m(0)}, l$ denotes the length of the subblock, and ε_c is the dielectric constant of the central plane ($\varepsilon_g(y = 0)$). In this research, we determined the corresponding parameters as $\lambda_0 = 1.55cm$, $\varepsilon_c = 3.0$, $d_m = d_0 = 3a$, $W = 51a$ and $l = 57a$. Here, a (≈ 0.4 cm) is the size of the unit that divides our metamaterial system into a series of small square grids. The input function is $E(y) = exp(-y^2/20)$.

In this work, the entire metamaterial (U0/GRIN/Um/GRIN) is limited in the y-direction by the width w, and in the x-direction by the total length d0+l+dm+l. Outsides the metamaterial, the material is defined as air (white area), and the width of the air region on each side is w/4. The simulated area is a three-dimensional (3D) rectangular region, which is non-uniformly gridded into slight meshes. The minimum mesh step is set to 50 nm, and the simulation time is set to 2000 fs so that the simulation results can be accurate. The periodic boundary was applied in the z-direction, while the perfectly matched layer-absorbing (PML) boundary was applied in the x- and y-directions toperform the simulation calculations.

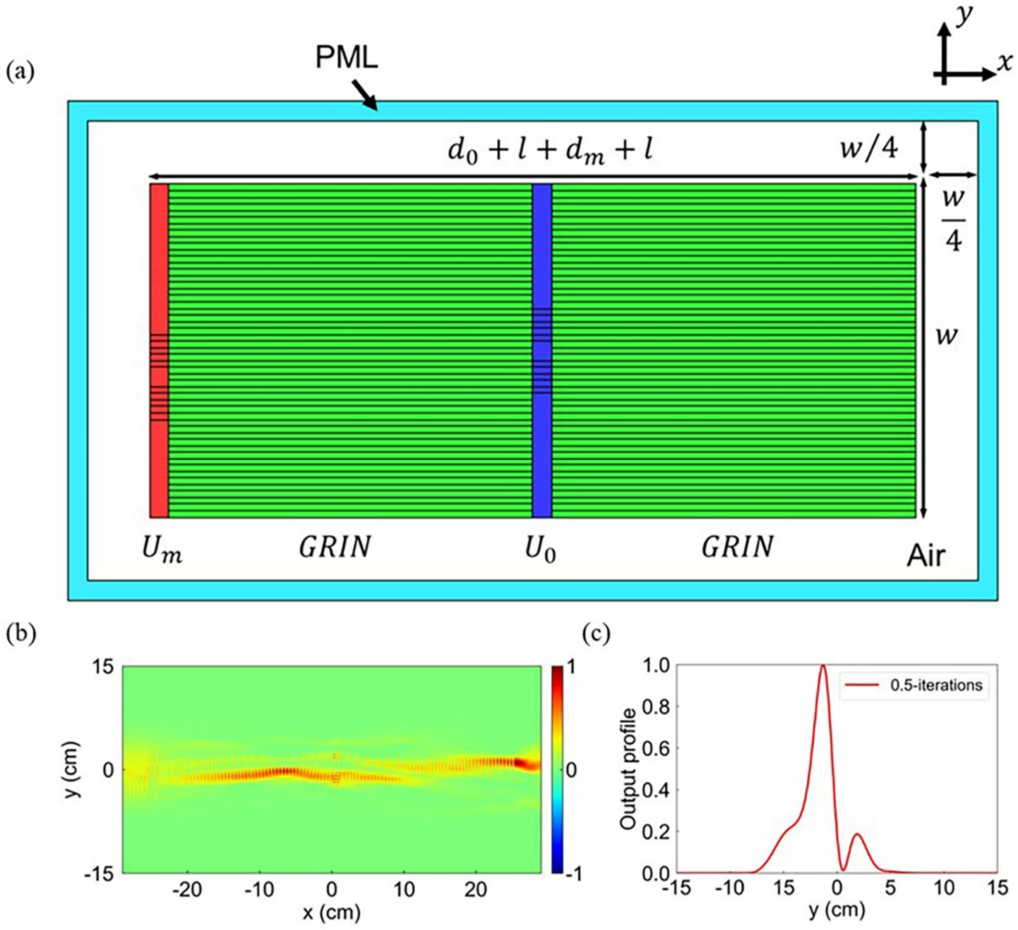

Figure S1. Theoretical simulation of the quantum search algorithm in free space. (**a**) Basic model built in Gallop. The designed metamaterial is comprised of four cascaded subblocks: an oracle subblock (red area, Um), two Fourier transform subblocks (green areas, GRID), and a phase plate subblock (blue area, U0). (**b**) The snapshots for the normalized intensity of the incident microwave throughout the metamaterial functioning as quantum searching simulator with 0.5-iterations. (**c**) Simulation results for the normalized output intensity of the metamaterial with the incident wave propagating 0.5-roundtrips within the metamaterial.

The snapshot of the electric field intensity of the whole system is shown in Figure S1b. In Figure S1c, we plot the distribution of the electric field intensity $|E(y)|^2$ at the output cross-section with 0.5-iterations. Several small side peaks are the result of diffraction effect during the iterations.
